# Inflammatory Mediators of Alzheimer’s Disease Characterized in a Mouse Model (APP/PS1)

**DOI:** 10.3390/neurosci7010023

**Published:** 2026-02-06

**Authors:** Adrian Jorda, Kenia Alvarez-Gamez, Ignacio Campo-Palacio, Juan Campos-Campos, Carlos Colmena, Sandeep Kumar Singh, Maria Jose Chiva Miralles, Constanza Aldasoro, Martin Aldasoro, Soraya L. Valles

**Affiliations:** 1 Department of Physiology, School of Medicine, University of Valencia, 46010 València, Spain; adrian.jorda@uv.es (A.J.); carcarmafisio@gmail.com (C.C.);; 2 Faculty of Nursing and Podiatry, University of Valencia, 46010 València, Spain; 3 Department of Medical Biotechnology, AIIMS, Nagpur 441108, MH, India

**Keywords:** Alzheimer, chemokines, inflammatory mediators, APP/PS1

## Abstract

Alzheimer’s disease (AD) is marked by amyloid plaques, hyperphosphorylated TAU proteins, and neuroinflammation. The APP/PS1 mouse model is widely used to study AD pathogenesis. In this study, we investigated the expression of chemokines and their receptors, which may play a role in AD’s pathological mechanisms, using brain cortex tissue from female APP/PS1 mice aged 20–21 months. We analyzed several chemokine receptors (CCR1, CCR2, CCR3, CCR4, CCR6, CCR7, CCR9, and CCR10) by Western blot and focused on CCR6, CCR7, and CCR10 using RT-PCR. Additionally, we quantified the levels of chemokines (CCL6, CCL8, CCL19, CCL20, CCL24, and CCL27) by RT-PCR. Our results showed a significant decrease in CCL8 and CCL19, along with their respective receptors, in the APP/PS1 mice compared to controls. On the other hand, we observed a notable increase in CCL6, CCL24, CCL20, CCL27, and their receptors. Chemokines like CCL8 and CCL20, involved in inflammatory responses, may reveal how neuroinflammation contributes to AD. CCL19 and CCL27 are linked to immune cell trafficking, which may help explain immune cell interactions with amyloid plaques and TAU tangles in the CNS. Overall, the altered expression of chemokines such as CCL24 could serve as biomarkers for early AD detection and monitoring disease progression. These findings suggest potential therapeutic targets to modulate immune responses and reduce neuroinflammation in AD.

## 1. Introduction

Alzheimer’s disease (AD) is a progressive neurodegenerative disorder and the most common form of dementia. It is characterized by cognitive impairments, particularly in memory and thinking, as well as changes in behavior and personality [1,2]. Key features of AD include extracellular accumulation of amyloid-beta (Aβ) plaques, hyperphosphorylation of TAU proteins, and glial activation, which are accompanied by synaptic dysfunction and neurodegeneration [3,4,5]. In the central nervous system (CNS), all types of neural cells participate in neuroinflammatory responses. Additionally, in experimental models induced by LPS (lipopolysaccharide), which triggers neuroimmune inflammation, an increase in the inflammatory response in the brain has been observed, contributing to the understanding of the neuroinflammation mechanisms in Alzheimer’s disease (AD) [6,7,8]. Several RNA sequencing datasets, such as GSE149661 and GSE145907, have provided an unbiased view of the expression of chemokines and their receptors in different neuroinflammatory conditions. These resources offer valuable information that can be used as a reference to compare and validate findings obtained from experimental AD models. In this study, we primarily focus on the APP/PS1 model, as it more faithfully represents key aspects of the disease, such as Aβ accumulation and TAU dysfunction, providing a relevant platform to explore alterations in the expression of chemokines and their receptors.

Amyloid-beta (Aβ) deposition has been associated with brain injuries, where cytokines and chemokines play a pivotal role. As key regulators of immune cell migration, chemokines are involved in various inflammatory processes that contribute to apoptosis and neurodegeneration [9]. Additionally, chemokines are implicated in neurodegeneration, inflammatory responses, oxidative stress, and programmed cell death pathways, particularly in Alzheimer’s disease (AD) [10,11,12]. CCL1, through its interaction with the CCR8 receptor, has been shown to reduce the clearance of Aβ peptides [13]. Both CCL3 and CCL4 are elevated in various AD models, with high concentrations found in microglia, astrocytes, and perivascular macrophages [9,14]. CCL4 and CCL2 are also linked to increased Aβ deposition [9]. CXCL10 is co-localized with Aβ in AD and has been associated with exacerbated TAU pathology [15,16]. Furthermore, CCL1 is essential in neuroimmunological interactions, serving as a bridge between neurons and microglia [9]. CCL3 and CCL4 have been identified in multiple AD models [9,17]. It is known that CCL3 and CCL6 induce cellular senescence, alongside increased expression of CDKI (cyclin-dependent kinase inhibitors), which are associated with microglial autophagy in neurodegenerative diseases characterized by extracellular amyloid accumulation [18,19]. Moreover, CXCL8 and CXCL12 have been detected in the cerebrospinal fluid (CSF) and plasma of AD patients [20]. Over recent decades, the interplay between AD pathology and neuroinflammation, particularly the gradual activation of astrocytes and microglia, has been a focus of research, also highlighting the roles of CCL19, CCL20, CCL24, and CCL27 [21]. The release of proinflammatory mediators, such as cytokines, chemokines, and reactive oxygen species (ROS), results in persistent activation of brain immune cells, promoting inflammation and mutual activation of microglia and astrocytes. This, in turn, triggers neuronal death [8] and increases Aβ accumulation. These mediators stimulate chemotaxis within the brain and cause cell death, affecting not only neurons but also oligodendrocytes and astrocytes [20].

The inflammatory process in Alzheimer’s disease (AD) is driven by the activation of microglia and astrocytes, which induce the production of molecular mediators that promote Aβ aggregation, neuronal and oligodendroglial loss, and synaptic damage. These inflammatory molecules contribute to the progression of neurodegeneration. Significant alterations in the levels of chemokines and their receptors have been observed in the brain, serum, and cerebrospinal fluid (CSF) in AD, leading to the initiation of an inflammatory cascade [9]. In this study, most chemokines and their receptors are predominantly expressed in microglia, which underlines their crucial role in the neuroinflammation processes observed in experimental models of AD. The aim of this study was to assess the protein expression of various inflammatory markers in the APP/PS1 mouse model, which is widely recognized for its relevance in studying neurodegeneration in AD. One such marker, CCR2, is expressed on microglia, where it promotes cell accumulation at the site of inflammation and enhances Aβ clearance [21]. Therefore, downregulation of CCR2 on microglia is associated with reduced Aβ clearance and increased mortality. Additionally, it is crucial to investigate the expression of CCR1, as this receptor mediates the signaling of major proinflammatory cytokines, such as IL-1, IL-6, and TNF-α. In this manuscript, we aim to assess as many inflammatory receptor proteins as possible using Western blotting and RT-PCR techniques. The proposed research on the chemokines CCL6, CCL8, CCL19, CCL20, CCL24, CCL27, and their corresponding receptors holds significant potential for advancing our understanding of Alzheimer’s disease (AD). The inclusion of CCL6, CCL24, CCL27, and their receptors in the context of Alzheimer’s disease is a novel contribution, as these factors have not been sufficiently studied in relation to neuroinflammation in this disease. Furthermore, the use of the APP/PS1 model allows for exploring the impact of these factors on disease progression, representing a significant advance in the field.

The importance of this research lies in several key areas:

(1) Investigating the role of the immune system in AD could help elucidate how immune responses contribute to disease progression. It may also reveal whether these chemokines could serve as potential biomarkers or therapeutic targets for neuroinflammation. Chemokines such as CCL8 and CCL20 are known to mediate inflammatory responses, and studying their involvement could provide valuable insight into how inflammation drives disease progression.
(2) Understanding immune cell recruitment is another critical aspect. CCL19 and CCL27 play essential roles in immune cell trafficking, and their involvement in the central nervous system (CNS) could offer new perspectives on how immune cells interact with amyloid plaques and TAU tangles.
(3) Dysregulation of chemokines like CCL24 may serve as biomarkers for the early detection or monitoring of disease progression in AD.
(4) By exploring the functions of these chemokines, researchers could identify novel therapeutic targets to modulate the immune response and reduce neuroinflammation, potentially slowing or halting the progression of AD.

## 2. Materials and Methods

### 2.1. Animals

APPswe/PS1 double-transgenic mice (B6C3-Tg) and their wild-type (WT) littermates were used in this study. The APP/PS1 mice carry a chimeric mouse/human APP695 cDNA with the Swedish (KM670/671NL) mutation, along with the human presenilin-1 (PS1) gene. Two groups were studied: APP/PS1 (*n* = 5) and WT (*n* = 5). The mice were given ad libitum access to a standard diet and were housed under a 12 h light/dark cycle at 22 °C. Genotyping was performed at 21–28 days of age. All procedures adhered to European regulations (CEE 86/609) and were approved by the University of Valencia Ethics Committee. Female brains were collected at 20–22 months of age, and the cortex was used for the experiments. The APP/PS1 mice used in this study were aged 20 to 22 months, a stage at which advanced Alzheimer’s pathology is expected, including high β-amyloid plaque burden and microglial activation. This age range was selected to study neuroinflammation at a late stage of the disease, minimizing variability associated with younger or much older ages. For the Western blot experiments, a small fraction of the cerebral cortex from each mouse hemisphere was used, approximately a few milligrams of tissue. Protein and RNA extraction were performed from a section of each cerebral hemisphere, while the remaining tissue was frozen for use in further experiments. Furthermore, our findings are limited to late-stage neuroinflammatory processes and do not address early detection or disease progression. Future studies that include younger age groups are needed to explore early-stage changes and AD progression.

### 2.2. Real-Time Polymerase Chain Reaction Analyses (RT-PCR)

Cortical brain tissue from each mouse was collected and stored in RNAlater solution (Ambion, Austin, TX, USA). RNA was extracted using TRIzol reagent (Thermo Fisher Scientific, Waltham, MA, USA), and its concentration and quality were assessed using RNA 6000 Nano LabChips on the Agilent 2100 Bioanalyzer (Agilent Technologies, Foster City, CA, USA). To quantify specific target genes, ready-to-use primers and probes from Thermo Fisher Scientific’s assay-on-demand service were used. The following primers were obtained from Thermo Fisher Scientific: CCL6 (Mm01302419_m1), CCL8 (Mm01297183_m1), CCL24 (Mm00444701_m1), CCL20 (Mm01268754_m1), CCL19 (Mm00839967_g1), CCL27a (Mm00441257_g1), CCR6 (Mm99999114_s1), CCR7 (Mm99999130_s1), CCR10 (Mm01946242_m1), and the endogenous reference gene β-actin (Mm00607939_s1). RNA samples were reverse transcribed using random hexamers and MultiScribe reverse transcriptase (Applied Biosystems, Waltham, MA, USA). Following complementary DNA (cDNA) synthesis, real-time polymerase chain reaction (RT-PCR) was performed using the ABI Prism 7900HT Sequence Detection System (Applied Biosystems). Samples were processed in triplicate, and expression variations were calculated using the 2^−ΔΔCt^ method.

### 2.3. Western Blot Analysis

Cortical protein extracts were mixed with SDS buffer, heated for 5 min, and quantified using a modified Lowry assay. Equal protein amounts were separated by SDS-PAGE and transferred to nitrocellulose membranes. After blocking with 5% milk in TBS-T, membranes were incubated with primary antibodies, followed by HRP-conjugated secondary antibodies (Cell Signaling Technologies, Barcelona, Spain). Immunoreactive bands were visualized using enhanced chemiluminescence (ECL; Pharmacia Biotech, San Francisco, CA, USA) and quantified with a Bio-Rad densitometer. The following antibodies were used: monoclonal anti-CCR2 (ab203128), anti-CCR1 (ab205719), anti-CCR4 (ab275980), anti-CCR3 (ab32512), anti-CCR9 (ab32556), anti-CCR6 (ab273580), anti-CCR7 (ab32075), anti-CCR10 (ab125224), and anti-tubulin (ab6046), all from Abcam Biotechnology (Madrid, Spain). See Supplementary Figure S1.

### 2.4. Data Analysis and Statistics

All data are expressed as mean ± standard deviation (SD) of independent biological replicates. Five independent biological replicates (individual mice per group) were included in the analysis. For each biological replicate, measurements from an average of four technical replicates were first averaged to obtain a single value per animal. Statistical differences between transgenic (APP/PS1) and wild-type (WT) mice were evaluated using an unpaired Student’s *t*-test. Statistical analyses were performed using GraphPad Prism software (version 10.6.0, GraphPad Software Inc., San Diego, CA, USA). A *p*-value < 0.05 was considered statistically significant.

## 3. Results

### 3.1. CCL6 Chemokine and Its Receptor CCR1

**Figure 1:**
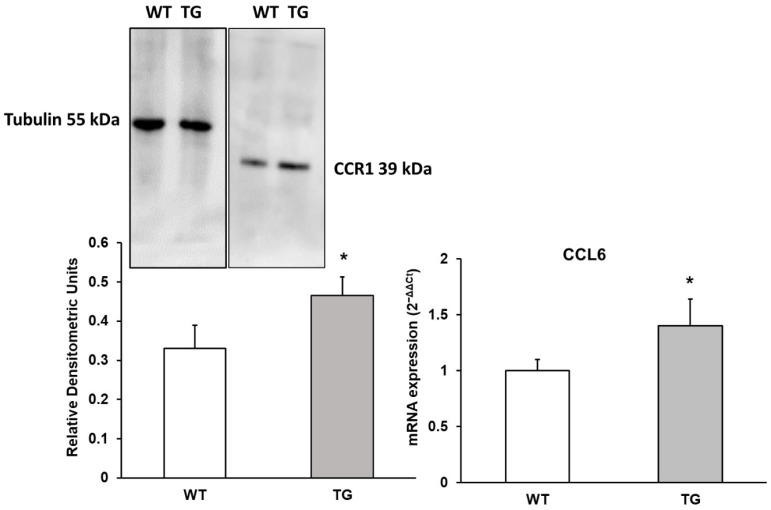
Protein expression of CCR1 and mRNA expression of CCL6 in the cortex of transgenic (TG) and wild-type (WT) mice. Data are presented as relative densitometric units (Western blot) and relative mRNA expression (2^−ΔΔCt^). Values represent the mean ± SD of five independent biological replicates (*n* = 5 per group), with each data point corresponding to the average of technical replicates. * *p* < 0.05 vs. WT.

CCL6 is a chemotactic factor that primarily attracts macrophages, but it can also recruit B cells, CD4+ lymphocytes, and eosinophils [22]. This chemokine binds to CCR1 (C-C Motif Chemokine Receptor 1), which is involved in regulating stem cell proliferation [23]. Changes in CCL6 expression, as well as alterations in its receptor expression, were assessed by RNA analysis and Western blotting. As shown in Figure 1, a significant 4.4-fold increase in CCL6 expression (140.1% ± 30%, *p* < 0.05) and approximately a 1.41-fold increase in its receptor CCR1 expression (140.91% ± 13.64%, *p* < 0.05) were observed in the APP/PS1 cerebral cortex compared to WT animals (Figure 1). These results suggest an upregulation of both the receptor and its associated chemokine, potentially linked to an increase in chemotaxis, given the role that CCR1 plays in this process.

### 3.2. CCL8 Chemokine and Its Receptor CCR2

**Figure 2:**
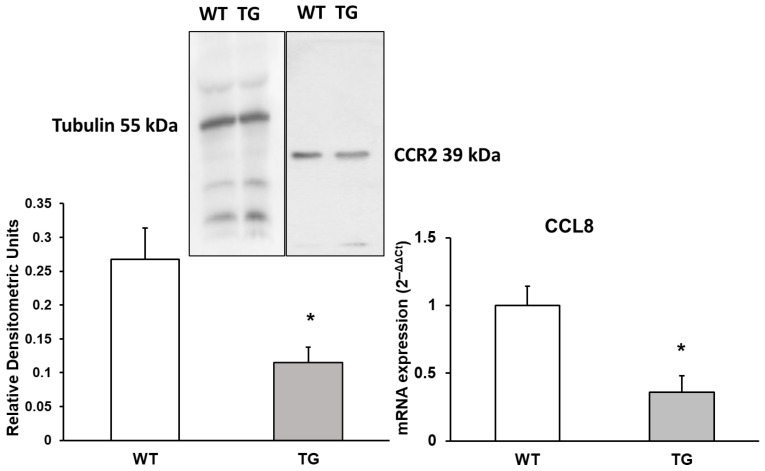
Protein expression of CCR2 and mRNA expression of CCL8 in the cortex of transgenic (TG) and wild-type (WT) mice. Data are presented as relative densitometric units (Western blot) and relative mRNA expression (2^−ΔΔCt^). Values represent the mean ± SD of five independent biological replicates (*n* = 5 per group), with each data point corresponding to the average of technical replicates. * *p* < 0.05 vs. WT.

CCL8 is a chemotactic factor that recruits monocytes and can promote endothelial cell migration, proliferation, and angiogenesis via CCR2 (C-C Motif Chemokine Receptor 2) by activating the extracellular signal-regulated kinase (ERK ½) signaling pathway [24]. Changes in CCL8 RNA expression and its receptor levels were assessed using Western blotting. Our results show a significant decrease in CCL8 expression, approximately 2.77-fold lower (36% ± 6%, *p* < 0.05), and a reduction in CCR2 expression by 2.33-fold (42.99% ± 12.01%, *p* < 0.05) in APP/PS1 mice compared to WT mice (Figure 2).

### 3.3. Evaluation of CCL24 Chemokine and CCR3 Receptor

**Figure 3:**
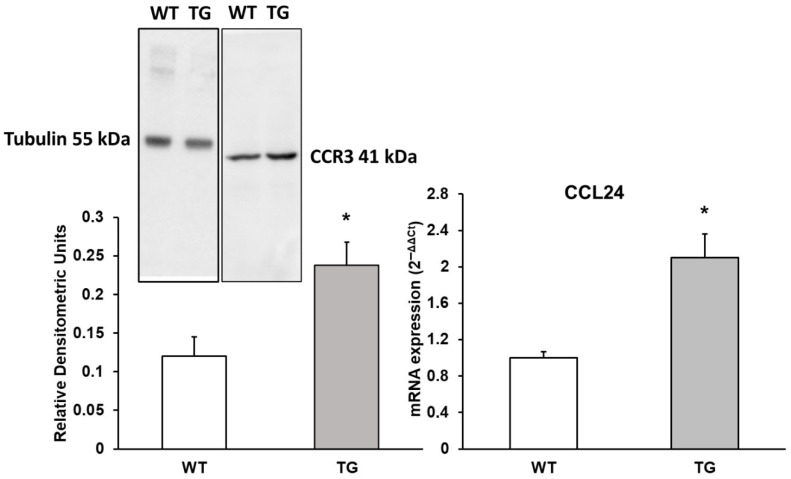
Protein expression of CCR3 and mRNA expression of CCL24 in the cortex of transgenic (TG) and wild-type (WT) mice. Data are presented as relative densitometric units (Western blot) and relative mRNA expression (2^−ΔΔCt^). Values represent the mean ± SD of five independent biological replicates (*n* = 5 per group), with each data point corresponding to the average of technical replicates. * *p* < 0.05 vs. WT.

CCL24 interacts with CCR3 (C-C Motif Chemokine Receptor 3) to induce chemotaxis in eosinophils [25]. As shown in Figure 3, there was a notable 2.1-fold increase in CCL24 RNA expression (210% ± 29%, *p* < 0.05) in APP/PS1 mice compared to WT mice. Additionally, its receptor CCR3 showed an approximately 1.98-fold increase in expression (197.91% ± 28.25%, *p* < 0.05) in APP/PS1 mice as measured by Western blot. The increase in both CCL24 and CCR3 expression may be related to enhanced chemotaxis processes (Figure 3).

### 3.4. CCR4 Receptor and CCR9 Receptor

**Figure 4:**
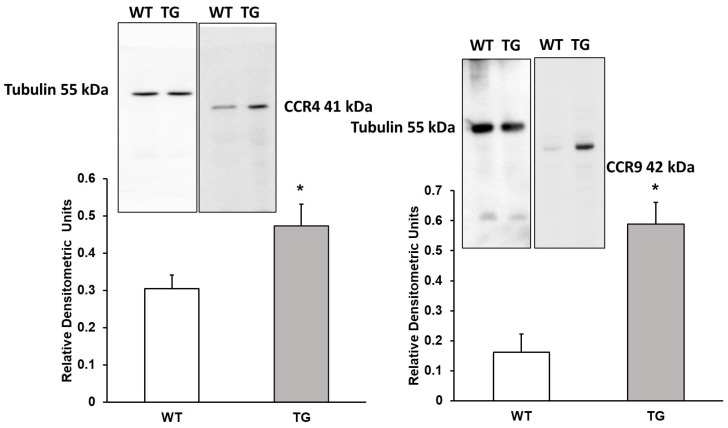
Protein expression of CCR4 and CCR9 receptors in the cortex of transgenic (TG) and wild-type (WT) mice. Data are presented as relative densitometric units (Western blot). Values represent the mean ± SD of five independent biological replicates (*n* = 5 per group), with each data point corresponding to the average of technical replicates. * *p* < 0.05 vs. WT.

CCR4 (C-C Motif Chemokine Receptor 4) may play a role in hippocampal neuron survival in the central nervous system (CNS). We observed a 1.55-fold increase in CCR4 expression (154.92% ± 22.13%, *p* < 0.05) in APP/PS1 mice compared to WT mice. These findings may be related to an increase in inflammatory processes in the CNS (Figure 4). Additionally, we assessed CCR9 (C-C Motif Chemokine Receptor 9) expression and detected a 3.61-fold increase (361.54% ± 44.61%, *p* < 0.05) in APP/PS1 mice compared to WT mice (Figure 4). Chemokines CCL4 and CCL25 were not included in this analysis, as the data were published previously [11].

### 3.5. CCL20 Chemokine and CCR6 Receptor

**Figure 5:**
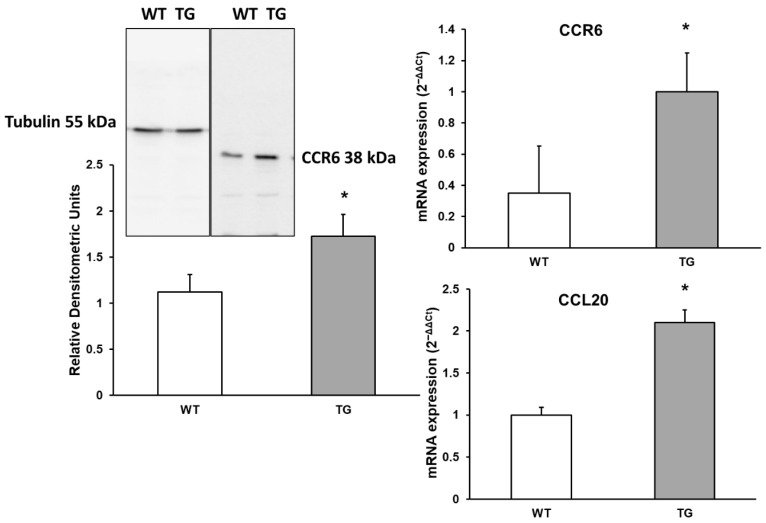
Protein expression of CCR6 and its chemokine CCL20 in the cortex of transgenic (TG) and wild-type (WT) mice. Data are presented as relative mRNA expression (2^−ΔΔCt^). Values represent the mean ± SD of five independent biological replicates (*n* = 5 per group), with each data point corresponding to the average of technical replicates. * *p* < 0.05 vs. WT.

CCL20 acts as a ligand for CCR6 (C-C Motif Chemokine Receptor 6). The CCL20–CCR6 ligand–receptor pair is crucial for the chemotaxis of dendritic cells, effector/memory T cells, and B cells. This pathway plays a significant role in skin and mucosal surfaces under both homeostatic and inflammatory conditions, as well as in pathological processes such as cancer and autoimmune diseases [26]. Changes in the expression of CCL20 and its receptor CCR6 were assessed. As shown in Figure 5, a significant 1.9-fold increase in CCL20 expression (210.1% ± 15.1%, *p* < 0.05) was detected in APP/PS1 mice compared to WT mice. Additionally, CCR6 expression showed an approximately 1.54-fold increase by Western blot (157.27% ± 20.4%, *p* < 0.05) and a 2.1-fold increase by RT-PCR (285.71% ± 71.43%, *p* < 0.05) in APP/PS1 mice compared to WT mice.

### 3.6. CCL19 Chemokine and CCR7 Receptor

**Figure 6:**
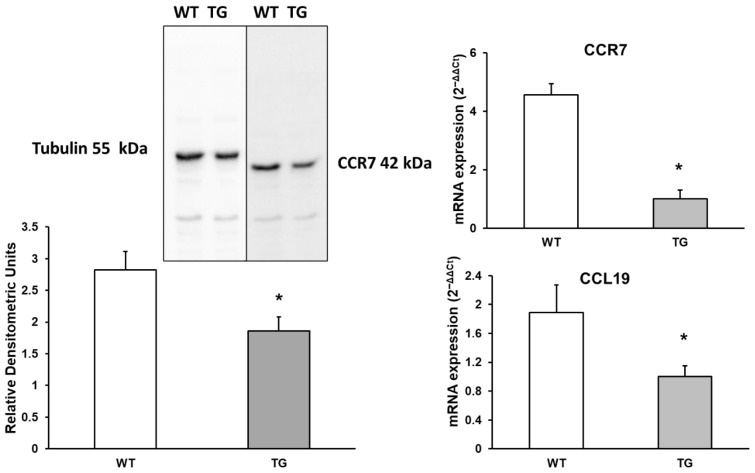
Protein expression of CCR7 and its chemokine CCL19 in the cortex of transgenic (TG) and wild-type (WT) mice. Data are presented as relative mRNA expression (2^−ΔΔCt^). Values represent the mean ± SD of five independent biological replicates (*n* = 5 per group), with each data point corresponding to the average of technical replicates. * *p* < 0.05 vs. WT.

CCL19 is a potent chemotactic factor for D4 T cells and CD8 T cells. It may also facilitate interactions between recirculating T cells and dendritic cells, as well as promote the migration of activated B cells into the T-zone of secondary lymphoid tissues [27]. This chemokine binds to CCR7 (C-C Motif Chemokine Receptor 7). mRNA expression of CCL19 and its receptor CCR7 was assessed using Western blot and RT-PCR. We observed a 1.89-fold decrease in CCL19 expression (189% ± 41%, *p* < 0.05) and a 1.52-fold decrease in CCR7 expression (152.43% ± 38%, *p* < 0.05) by Western blot. Additionally, a significant 4.56-fold decrease in CCR7 expression was observed by RT-PCR (456% ± 95%, *p* < 0.05) in APP/PS1 mice compared to WT mice (Figure 6).

### 3.7. CCL27 Chemokine and CCR10 Receptor

**Figure 7:**
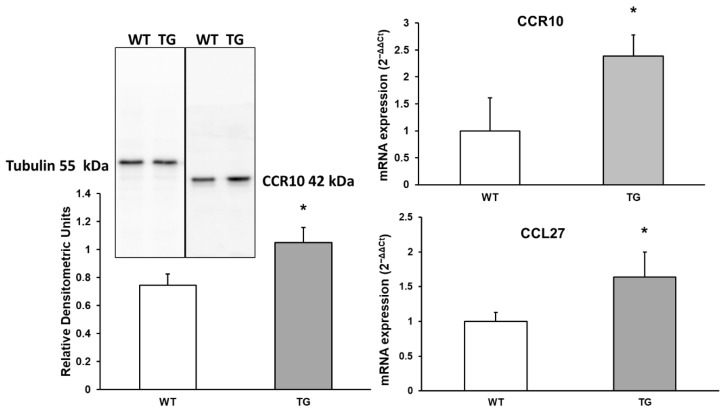
Protein expression of CCR10 and its chemokine CCL27 in the cortex of transgenic (TG) and wild-type (WT) mice. Data are presented as relative mRNA expression (2^−ΔΔCt^). Values represent the mean ± SD of five independent biological replicates (*n* = 5 per group), with each data point corresponding to the average of technical replicates. * *p* < 0.05 vs. WT.

CCL27 is a chemotactic factor that attracts skin-associated memory T lymphocytes and may play a role in directing lymphocytes to cutaneous sites [28]. This chemokine binds to CCR10, and changes in its expression were assessed using Western blot and RT-PCR. As shown in Figure 7, a 1.64-fold increase in CCL27 expression was observed in APP/PS1 mice compared to WT mice (164% ± 46%, *p* < 0.05). Additionally, a significant 1.41-fold increase in CCR10 expression (141.49% ± 17.95%, *p* < 0.05) was detected by Western blot, and an approximately 2.39-fold increase by RT-PCR (239.05% ± 16.1%, *p* < 0.05) in APP/PS1 mice compared to WT mice (Figure 7).

## 4. Discussion

Our results demonstrate alterations in the expression of chemokines and their receptors, which may trigger processes such as chemotaxis, inflammation, phagocytosis, and demyelination in APP/PS1 transgenic mice compared to WT animals (Table 1). Protein expression of chemokine receptors CCR1, CCR3, CCR4, CCR6, CCR9, and CCR10 was higher in transgenic mice compared to WT, whereas CCR2 and CCR7 protein expression was reduced in APP/PS1 mice relative to WT. The central nervous system (CNS) expresses different chemokine receptors depending on the cell type in Alzheimer’s disease (AD) [29]. Neurons express CCR1, CCR2, CCR3, CCR5, and CCR10 [30,31]. CCL2 and CCL19 are expressed constitutively under normal physiological conditions [32,33], while other chemokines exhibit altered expression during inflammation. Additionally, some chemokines, such as CCL20 and CCR6, change their expression in neurodegenerative conditions like AD [34], while others, such as CCL27 and CCR10, are upregulated in cancerous cells [35].

While altered levels of chemokines such as CCL5, CCL15, IP-10, and MCP-1 in blood and cerebrospinal fluid (CSF) have been previously identified as potential diagnostic biomarkers for Alzheimer’s disease (AD), the chemokines explored in this study—CCL6, CCL8, CCL24, CCL19, CCL20, and CCL27—have not been as widely studied in the context of AD. These chemokines are implicated in neuroinflammatory responses, but their specific roles in the pathogenesis of AD remain poorly understood. Our findings indicate that these chemokines and their receptors are altered in APP/PS1 transgenic mice, suggesting they could be involved in the neuroinflammatory processes characteristic of AD. This study highlights the novelty of investigating the expression of these lesser-known chemokines in the context of Alzheimer’s disease. We propose that targeting these chemokine pathways could offer new therapeutic avenues for modulating the immune response in AD. Specifically, strategies aimed at regulating CCL6, CCL8, CCL24, CCL19, CCL20, and CCL27 might reduce neuroinflammation, protect synaptic function, and potentially slow disease progression. Given their involvement in other inflammatory conditions and cancers, these chemokines could also represent broader therapeutic targets that extend beyond AD, opening new research opportunities for treatment strategies that address both neurodegenerative diseases and other inflammatory pathologies.

CCL6 is a cytokine from the chemokine family that primarily attracts macrophages, CD4+ lymphocytes, and eosinophils, but it can also recruit B cells [36]. It is expressed in neutrophils and macrophages and is induced following myeloid cell differentiation [36]. However, reduced expression has been reported in activated T cells [37]. CCR1 (C-C Motif Chemokine Receptor 1), which is involved in regulating stem cell proliferation [38] when bound to CCL6, plays a crucial role in the pathogenesis of IL-13-induced inflammation [39]. Increased IL-13 expression has been observed in APP/PS1 mice [9] compared to wild-type mice, highlighting the link between elevated IL-13 levels and increased CCR1 and CCL6 expression. In Alzheimer’s disease patients, CCR1 has been identified as a specific marker of the disease, independent of Aβ1-42 deposition [40].

CCR2 can bind to the chemokines CCL2, CCL7, CCL8, and CCL1 [11,37,41,42] and is expressed in neurons, astrocytes, and microglia [11,43,44]. CCR2 deficiency exacerbates cognitive impairment and β-amyloid deposition in animal models of Alzheimer’s disease [13,14]. We observed a decrease in CCR2 protein expression in APP/PS1 mice compared to WT mice, as detected by RT-PCR in a previously published study [11]. Both CCR2 and CCL8 are critical for maintaining neuronal homeostasis [11,44]. In CCR2-/- mice, a reduction in microglial cell survival has been noted, accompanied by increased Aβ deposits [45,46]. Additionally, CCR2 deficiency has been associated with the rapid progression of Alzheimer’s disease [45]. CCL8 is a chemokine that binds to CCR1, CCR2, CCR3, and CCR5 [47,48]. This chemokine recruits various immune cells, including mast cells, basophils, eosinophils, monocytes, T cells, and NK cells, all of which play roles in inflammation and allergic responses [49].

CCL24, also known as myeloid progenitor inhibitory factor 2 (MPIF-2), is located on human chromosome 7 and interacts with the CCR3 receptor chemokine [50,51]. CCL24 induces chemotaxis in eosinophils [52] and resting T lymphocytes, with a weaker attraction for neutrophils. CCR3, in turn, can bind and respond to various chemokines, including CCL11, CCL26, CCL7, CCL13, CCL24, and CCL5. The receptor is highly expressed in basophils and eosinophils [52], and both CCR3 and CCR5 have been shown to facilitate HIV-1 infection [53] by allowing viral entry into cells that also express CD4, the primary HIV-1 receptor [53]. In Alzheimer’s disease (AD) patients, CCR3 is expressed in hippocampal neurons. Treatment of primary hippocampal neurons with CCL11 results in the activation of cyclin-dependent kinase 5 (CDK5) and glycogen synthase kinase-3β (GSK3β), leading to increased TAU phosphorylation [48]. In our study, we observed an increase in CCR3 and its chemokine CCL24 in APP/PS1 transgenic mice, as previously reported in our RT-PCR analysis [11]. The deletion of CCR3 in APP/PS1 mice significantly reduced CDK5 and GSK3β phosphorylation, TAU hyperphosphorylation, Aβ deposition, microgliosis, astrogliosis, synaptic loss, and deficits in spatial learning and memory. Therefore, the age-related increase in CCL24 may provide therapeutic benefits for AD, and antagonizing CCR3 could be a promising approach for treating the disease [54].

Figure 4 shows that CCR4 protein expression, as detected by Western blot, is elevated in APP/PS1 mice compared to wild-type mice. This receptor binds to the chemokines CCL4, CCL17, and CCL22 [11,50,55] and is expressed in neurons and astrocytes [56]. In transgenic mice, where CCL17 chemokine synthesis was eliminated, improved CNS immune responses were observed, including enhanced Aβ clearance and reduced neuronal death. CCR4 expression is increased in cells involved in defense mechanisms, making it an intriguing target for the treatment of various immunological diseases [57,58].

CCR6 binds to the CCL20 chemokine and has been identified as a biomarker for Alzheimer’s disease (AD) in a triple transgenic mouse model [35]. This protein is part of the family A G-protein-coupled receptor superfamily [59]. CCR6 is expressed on B cells, T cells, natural killer T cells, and neutrophils [11]. CCL20, also known as macrophage inflammatory protein 3α, is the sole chemokine for this receptor [12]. Interleukin-4 (IL-4) and interferon-γ can reduce CCR6 expression during Langerhans cell development, while IL-10 increases its expression [60,61]. The CCR6-CCL20 axis has been implicated in several types of cancer, including hepatocellular carcinoma, colorectal cancer, breast cancer, pancreatic cancer, cervical cancer, and kidney cancer. This axis promotes cancer progression by enhancing the migration and proliferation of cancer cells [61].

Our results show an increase in CCR9 protein expression in APP/PS1 mice. The cytokine CCL25, which binds to CCR9, was assessed by RT-PCR in our previous study [11]. Both CCR9 and CCL25 are expressed in the hippocampus, cortex, and cerebellum [62]. CCL25 levels are elevated in patients with mild cognitive impairment (prior to AD) and those experiencing cognitive decline [62]. CCL25 is also implicated in the etiology of ulcerative colitis [63] and other inflammatory bowel diseases [64]. CCR9 antagonists have been shown to reduce cytokines involved in Crohn’s disease [65]. Additionally, CCL25 and CCR9 reduce beta-cell functionality, inhibiting insulin release. Inhibition of CCL25 or blockage of CCR9 may offer potential treatments for type 2 diabetes [66]. Recently, several studies have linked Alzheimer’s disease to a specific form of diabetes known as type 3 diabetes [67]. In this condition, insulin resistance occurs alongside the development of AD [67].

CCR7 is a chemokine receptor whose deficiency has been linked to aging, cognitive impairment, and neuroinflammation [68]. Lack of CCR7 results in changes to the cerebral vascular endothelium, which, along with a reduction in AQP4 levels, leads to impaired cerebral lymphatic drainage mechanisms [68]. Moreover, decreased CCR7 function has been implicated in several pathophysiological processes in Alzheimer’s disease (AD), including TAU hyperphosphorylation, β-amyloid pathology, and neuronal death. CCR7-knockout mice show worsened cognition, emotional behavior, and sociability [69]. CCL19, a homeostatic chemokine, is abundantly expressed in the thymus and lymph nodes. It primarily regulates immune cell trafficking and plays a role in cancer development [70]. However, CCL19 also acts as a tumor-supportive factor by promoting inflammation, cell growth, and metastasis [70]. Several proteins, including IL-10, LIF-R, TWEAK, CCL19, IL-17C, MCP-4, and TGF-alpha, have been associated with multiple cognitive domains and the risk of developing dementia [70]. CCL19 is present in normal cerebral tissue, where it modulates inflammatory processes and aids in the repair of multiple sclerosis lesions [71]. Our study demonstrates a significant increase in the expression of both CCR10 and CCL27 in transgenic mice compared to wild-type (WT) mice. CCR10 expression is elevated in melanoma, and its interaction with CCL27 promotes rapid tumor progression [72]. The CCR10/CCL27 interaction also enhances metastasis in various types of lung cancer via the PI3K-Akt signaling pathway [73]. Furthermore, CCR10 plays a role in the development of Alzheimer’s disease (AD) [71]. CCL27 has been linked to the progression of Alzheimer’s and cognitive decline [71], and it has been identified as a potential marker in the processes leading to cognitive deterioration [72].

It is important to note that this study does not determine the specific cellular origin of the chemokine receptors, as the analyses were performed on cortical homogenates. The inferences about expression in different cell types are based on the existing literature. Future studies using techniques such as immunofluorescence or flow cytometry would be needed to accurately identify the cellular profiles of these receptors. Although aspects of cancer biology related to CCR6 and CCR10 are mentioned, their inclusion aims to provide a broader context regarding the mechanisms of these receptors, which are involved in both neuroinflammation in Alzheimer’s disease (AD) and other pathologies. Parallels between these fields can offer additional insights into their role in modulating the immune response in AD. This study used only female mice, which may limit the interpretation of the results due to sex differences in Alzheimer’s pathology and inflammatory signaling. Including both sexes in future studies would be important to assess potential differences in disease progression and immune response.

## 5. Conclusions

Our results indicate that changes in the expression of various chemokines and their receptors in APP/PS1 transgenic mice may play a crucial role in cognitive impairment characteristic of Alzheimer’s disease (AD). Specifically, altered expression of the CCR1–CCL6, CCR3–CCL24, CCR4–CCL4, CCR6–CCL20, CCR9–CCL25, and CCR10–CCL27 pairs, together with the downregulation of CCR2–CCL8 and CCR7–CCL19, points to a potential dysreg-ulation of neuroinflammatory pathways that may contribute to AD pathology. These findings suggest that targeting specific chemokine receptors may provide new therapeutic avenues, such as modulating the immune response to reduce neuroinflammation, improving synaptic function, and potentially slowing disease progression. Additionally, the involvement of some of these chemokines in non-brain pathologies (e.g., CCR4 in immunological diseases, CCR9 in Crohn’s disease and type 2 diabetes, and CCR6/CCL20/CCR10/CCL27 in cancer) highlights the broader therapeutic potential of targeting these pathways in both AD and other inflammatory conditions (Table 1).

## Figures and Tables

**Table 1:** Summary of chemokine and chemokine receptor expression changes in the cerebral cortex of APP/PS1 mice compared to WT controls.

Chemokine	Receptor	Method	Change in APP/PS1 vs. WT	Fold Change (% of WT)	*p*-Value
CCL6	CCR1	RT-PCR/WB	Increased	CCL6: 4.4-fold (140.1 ± 30%) CCR1: 1.41-fold (140.9 ± 13.6%)	<0.05
CCL8	CCR2	RT-PCR/WB	Decreased	CCL8: 2.77-fold ↓ (36 ± 6%) CCR2: 2.33-fold ↓ (43.0 ± 12.0%)	<0.05
CCL24	CCR3	RT-PCR/WB	Increased	CCL24: 2.1-fold (210 ± 29%) CCR3: 1.98-fold (197.9 ± 28.3%)	<0.05
–	CCR4	WB	Increased	CCR4: 1.55-fold (154.9 ± 22.1%)	<0.05
–	CCR9	WB	Increased	CCR9: 3.61-fold (361.5 ± 44.6%)	<0.05
CCL20	CCR6	RT-PCR/WB	Increased	CCL20: 1.9-fold (210.1 ± 15.1%) CCR6 WB: 1.54-fold (157.3 ± 20.4%) CCR6 RT-PCR: 2.1-fold (285.7 ± 71.4%)	<0.05
CCL19	CCR7	RT-PCR/WB	Decreased	CCL19: 1.89-fold ↓ (≈53%) CCR7 WB: 1.52-fold ↓ (≈66%) CCR7 RT-PCR: 4.56-fold ↓ (≈22%)	<0.05
CCL27	CCR10	RT-PCR/WB	Increased	CCL27: 1.64-fold (164 ± 46%) CCR10 WB: 1.41-fold (141.5 ± 18.0%) CCR10 RT-PCR: 2.39-fold (239.1 ± 16.1%)	<0.05

## Data Availability

The original contributions presented in this study are included in the article/Supplementary Material. Further inquiries can be directed to the corresponding author.

## Supplementary Materials

The following supporting information can be downloaded at: https://www.mdpi.com/article/10.3390/neurosci7010023/s1, Figure S1: Representative Western blot analyses of CCR1, CCR2, CCR3, CCR4, CCR9, CCR6, CCR7 and CCR10 in Wild and trans-genic mice. Multiple blots for each protein are shown to demonstrate experimental reproducibility. Tubuline was used as a loading control. Molecular weight markers are indicated.

## References

[1] Liu C.C., Wang N., Chen Y., Inoue Y., Shue F., Ren Y., Wang M., Qiao W., Ikezu T.C., Li Z. (2023). Cell-autonomous effects of APOE4 in restricting microglial response in brain homeostasis and Alzheimer’s disease. Nat. Immunol..

[2] Giunta B., Fernandez F., Nikolic W.V., Obrego E., Rrapo E., Town T., Tan J. (2008). Inflammaging as a prodrome to Alzheimer’s disease. J. Neuroinflamm..

[3] Wyss-Coray T. (2006). Inflammation in Alzheimer disease: Driving force, bystander, or beneficial response?. Nat. Med..

[4] Reitz C., Mayeux R. (2014). Alzheimer disease: Epidemiology, diagnostic criteria, risk factors and biomarkers. Biochem. Pharmacol..

[5] Twarowski B., Herbet M. (2023). Inflammatory Processes in Alzheimer’s Disease-Pathomechanism, Diagnosis and Treatment: A Review. Int. J. Mol. Sci..

[6] Blanco A.M., Vallés S.L., Pascual M., Guerri C. (2005). Involvement of TLR4/type I IL-1 receptor signaling in the induction of inflammatory mediators and cell death induced by ethanol in cultured astrocytes. J. Immunol..

[7] DiSabato D.J., Quan N., Godbout J.P. (2016). Neuroinflammation: The devil is in the details. J. Neurochem..

[8] Thakur S., Dhapola R., Sarma P., Medhi B., Reddy D.H. (2023). Neuroinflammation in Alzheimer’s Disease: Current Progress in Molecular Signaling and Therapeutics. Inflammation.

[9] Jorda A., Cauli O., Santonja J.M., Aldasoro M., Aldasoro C., Obrador E., Vila J.M., Mauricio M.D., Iradi A., Guerra-Ojeda S. (2019). Changes in Chemokines and Chemokine Receptors Expression in a Mouse Model of Alzheimer’s Disease. Int. J. Biol. Sci..

[10] Moylan S., Berk M., Dean O.M., Samuni Y., Williams L.J., O’Neil A., Hayley A.C., Pasco J.A., Anderson G., Jacka F.N. (2014). Oxidative & nitrosative stress in depression: Why so much stress?. Neurosci. Biobehav. Rev..

[11] Jorda A., Aldasoro M., Aldasoro C., Valles S.L. (2021). Inflammatory Chemokines Expression Variations and Their Receptors in APP/PS1 Mice. J. Alzheimers Dis..

[12] Jorda A., Campos-Campos J., Iradi A., Aldasoro M., Aldasoro C., Vila J.M., Valles S.L. (2020). The Role of Chemokines in Alzheimer’s Disease. Endocr. Metab. Immune Disord. Drug Targets.

[13] Puntambekar S.S., Moutinho M., Lin P.B., Jadhav V., Tumbleson-Brink D., Balaji A., Benito M.A., Xu G., Oblak A., Lasagna-Reeves C.A. (2022). CX3CR1 deficiency aggravates amyloid driven neuronal pathology and cognitive decline in Alzheimer’s disease. Mol. Neurodegener..

[14] Takata K., Amamiya T., Mizoguchi H., Kawanishi S., Kuroda E., Kitamura R., Ito A., Saito Y., Tawa M., Nagasawa T. (2018). Alpha7 nicotinic acetylcholine receptor-specific agonist DMXBA (GTS-21) attenuates Aβ accumulation through suppression of neuronal γ-secretase activity and promotion of microglial amyloid-β phagocytosis and ameliorates cognitive impairment in a mouse model of Alzheimer’s disease. Neurobiol. Aging.

[15] Walker D.G., Lue L.F., Beach T.G. (2001). Gene expression profiling of amyloid beta peptide-stimulated human post-mortem brain microglia. Neurobiol. Aging.

[16] Naert G., Rivest S. (2011). CC chemokine receptor 2 deficiency aggravates cognitive impairments and amyloid pathology in a transgenic mouse model of Alzheimer’s disease. J. Neurosci..

[17] Naert S., Rivest A. (2013). Deficiency in CCR2 + monocytes: The hidden side of Alzheimer’s disease. J. Mol. Cell Biol..

[18] Zhu M., Allard J.S., Zhang Y., Perez E., Spangler E.L., Becker K.G., Rapp P.R. (2014). Age-related brain expression and regulation of the chemokine CCL4/MIP-1β in APP/PS1 double-transgenic mice. J. Neuropathol. Exp. Neurol..

[19] Choi I., Wang M., Yoo S., Xu P., Seegobin S.P., Li X., Han X., Wang Q., Peng J., Zhang B. (2023). Autophagy enables microglia to engage amyloid plaques and prevents microglial senescence. Nat. Cell Biol..

[20] Nie J., Fang Y., Chen Y., Aidina A., Qiu Q., Zhao L., Liu X., Sun L., Li Y., Zhong C. (2022). Characteristics of Dysregulated Proinflammatory Cytokines and Cognitive Dysfunction in Late-Life Depression and Amnestic Mild Cognitive Impairment. Front. Immunol..

[21] Hickman S.E., Khoury J.E. (2010). Mechanisms of mononuclear phagocyte recruitment in Alzheimer’s disease. CNS Neurol. Disord. Drug Targets.

[22] Du X., Li F., Zhang C., Li N., Huang H., Shao Z., Zhang M., Zhan X., He Y., Ju Z. (2021). Eosinophil-derived chemokine (hCCL15/23, mCCL6) interacts with CCR1 to promote eosinophilic airway inflammation. Signal Transduct. Target. Ther..

[23] Zeissig M.N., Hewett D.R., Panagopoulos V., Mrozik K.M., To L.B., Croucher P.I., Zannettino A.C.W., Vandyke K. (2021). Expression of the chemokine receptor CCR1 promotes the dissemination of multiple myeloma plasma cells in vivo. Haematologica.

[24] Ciechanowska A., Mika J. (2024). CC Chemokine Family Members’ Modulation as a Novel Approach for Treating Central Nervous System and Peripheral Nervous System Injury-A Review of Clinical and Experimental Findings. Int. J. Mol. Sci..

[25] Gela A., Kasetty G., Mörgelin M., Bergqvist A., Erjefält J.S., Pease J.E., Egesten A. (2016). Osteopontin binds and modulates functions of eosinophil-recruiting chemokines. Allergy.

[26] Kwantwi L.B., Boafo J.D., Egleh B.E., Li M. (2025). CCL20 in the tumor microenvironment: Implications for cancer progression and therapeutic approaches. Clin. Transl. Oncol..

[27] Brandum E.P., Jørgensen A.S., Rosenkilde M.M., Hjortø G.M. (2021). Dendritic Cells and CCR7 Expression: An Important Factor for Autoimmune Diseases, Chronic Inflammation, and Cancer. Int. J. Mol. Sci..

[28] Davila M.L., Xu M., Huang C., Gaddes E.R., Winter L., Cantorna M.T., Wang Y., Xiong N. (2022). CCL27 is a crucial regulator of immune homeostasis of the skin and mucosal tissues. iScience.

[29] Mecca C., Giambanco I., Donato R., Arcuri C. (2018). Microglia and Aging: The Role of the TREM2-DAP12 and CX3CL1-CX3CR1 Axes. Int. J. Mol. Sci..

[30] Jaerve A., Müller H.W. (2012). Chemokines in CNS injury and repair. Cell Tissue Res..

[31] Stuart M.J., Singhal G., Baune B.T. (2015). Systematic Review of the Neurobiological Relevance of Chemokines to Psychiatric Disorders. Front. Cell. Neurosci..

[32] Reaux-Le Goazigo A., Van Steenwinckel J., Rostène W., Mélik Parsadaniantz S. (2013). Current status of chemokines in the adult CNS. Prog. Neurobiol..

[33] Yamamoto M., Horiba M., Buescher J.L., Huang D., Gendelman H.E., Ransohoff R.M., Ikezu T. (2005). Overexpression of monocyte chemotactic protein-1/CCL2 in beta-amyloid precursor protein transgenic mice show accelerated diffuse beta-amyloid deposition. Am. J. Pathol..

[34] Subramanian S., Ayala P., Wadsworth T.L., Harris C.J., Vandenbark A.A., Quinn J.F., Offner H. (2010). CCR6: A biomarker for Alzheimer’s-like disease in a triple transgenic mouse model. J. Alzheimers Dis..

[35] Korbecki J., Grochans S., Gutowska I., Barczak K., Baranowska-Bosiacka I. (2020). CC Chemokines in a Tumor: A Review of Pro-Cancer and Anti-Cancer Properties of Receptors CCR5, CCR6, CCR7, CCR8, CCR9, and CCR10 Ligands. Int. J. Mol. Sci..

[36] Orlofsky A., Berger M.S., Prystowsky M.B. (1991). Novel expression pattern of a new member of the MIP-1 family of cytokine-like genes. Cell Regul..

[37] Ma B., Zhu Z., Homer R.J., Gerard C., Strieter R., Elias J.A. (2004). The C10/CCL6 chemokine and CCR1 play critical roles in the pathogenesis of IL-13-induced inflammation and remodeling. J. Immunol..

[38] Marques R.E., Guabiraba R., Russo R.C., Teixeira M.M. (2013). Targeting CCL5 in inflammation. Expert Opin. Ther. Targets.

[39] Banisadr G., Quéraud-Lesaux F., Boutterin M.C., Pélaprat D., Zalc B., Rostène W., Haour F., Parsadaniantz S.M. (2002). Distribution, cellular localization, and functional role of CCR2 chemokine receptors in adult rat brain. J. Neurochem..

[40] Tran P.B., Banisadr G., Ren D., Chenn A., Miller R.J. (2007). Chemokine receptor expression by neural progenitor cells in neurogenic regions of mouse brain. J. Comp. Neurol..

[41] Kiyota H.E., Gendelman R.A., Weir E.E., Higgins G., Zhang M., Jain M. (2013). CCL2 affects β-amyloidosis and progressive neurocognitive dysfunction in a mouse model of Alzheimer’s disease Neurobiol. Aging.

[42] Azizi G., Khannazer N., Mirshafiey A. (2014). The potential role of chemokines in Alzheimer’s disease pathogenesis. Am. J. Alzheimers Dis. Other Dement..

[43] Mildner A., Schlevogt B., Kierdorf K., Böttcher C., Erny D., Kummer M.P., Quinn M., Brück W., Bechmann I., Heneka M.T. (2011). Distinct and non-redundant roles of microglia and myeloid subsets in mouse models of Alzheimer’s disease. J. Neurosci..

[44] Philipson O., Lord A., Gumucio A., O’Callaghan P., Lannfelt L., Nilsson L.N. (2010). Animal models of amyloid-beta-related pathologies in Alzheimer’s disease. FEBS J..

[45] El Khoury J., Toft M., Hickman S.E., Means T.K., Terada K., Geula C., Luster A.D. (2007). Ccr2 deficiency impairs microglial accumulation and accelerates progression of Alzheimer-like disease. Nat. Med..

[46] Cedile O., Wlodarczyk A., Owens T. (2017). CCL2 recruits T cells into the brain in a CCR2-independent manner. APMIS.

[47] Ge B., Li J., Wei Z., Sun T., Song Y., Khan N.U. (2017). Functional expression of CCL8 and its interaction with chemokine receptor CCR3. BMC Immunol..

[48] Proost P., Wuyts A., Van Damme J. (1996). Human monocyte chemotactic proteins-2 and -3: Structural and functional comparison with MCP-1. J. Leukoc. Biol..

[49] Patel V.P., Kreider B.L., Li Y., Li H., Leung K., Salcedo T., Nardelli B., Pippalla V., Gentz S., Thotakura R. (1997). Molecular and functional characterization of two novel human C-C chemokines as inhibitors of two distinct classes of myeloid progenitors. J. Exp. Med..

[50] Hillier L.W., Fulton R.S., Fulton L.A., Graves T.A., Pepin K.H., Wagner-McPherson C., Layman D., Maas J., Jaeger S., Walker R. (2003). The DNA sequence of human chromosome 7. Nature.

[51] White J.R., Imburgia C., Dul E., Appelbaum E., O’Donnell K., O’Shannessy D.J., Brawner M., Fornwald J., Adamou J., Elshourbagy N.A. (1997). Cloning and functional characterization of a novel human CC chemokine that binds to the CCR3 receptor and activates human eosinophils. J. Leukoc. Biol..

[52] Klementowicz J.E., Mahne A.E., Spence A., Nguyen V., Satpathy A.T., Murphy K.M., Tang Q. (2017). Cutting Edge: Origins, Recruitment, and Regulation of CD11c+ Cells in Inflamed Islets of Autoimmune Diabetes Mice. J. Immunol..

[53] Choe H., Farzan M., Sun Y., Sullivan N., Rollins B., Ponath P.D., Wu L., Mackay C.R., LaRosa G., Newman W. (1996). The beta-chemokine receptors CCR3 and CCR5 facilitate infection by primary HIV-1 isolates. Cell.

[54] Zhu C., Xu B., Sun X., Zhu Q., Sui Y. (2017). Targeting CCR3 to Reduce Amyloid-β Production, TAU Hyperphosphorylation, and Synaptic Loss in a Mouse Model of Alzheimer’s Disease. Mol. Neurobiol..

[55] Kusumoto M., Xu B., Shi M., Matsuyama T., Aoyama K., Takeuchi T. (2007). Expression of chemokine receptor CCR4 and its ligands (CCL17 and CCL22) in murine contact hypersensitivity. J. Interferon Cytokine Res..

[56] Goldeck D., Larbi A., Pellicanó M., Alam I., Zerr I., Schmidt C., Fulop T., Pawelec G. (2013). Enhanced Chemokine Receptor Expression on Leukocytes of Patients with Alzheimer’s Disease. PLoS ONE.

[57] Cheng W., Zhao Q., Xi Y., Li C., Xu Y., Wang L., Niu X., Wang Z., Chen G. (2015). IFN-β inhibits T cells accumulation in the central nervous system by reducing the expression and activity of chemokines in experimental autoimmune encephalomyelitis. Mol. Immunol..

[58] Khaibullin T., Ivanova V., Martynova E., Cherepnev G., Khabirov F., Granatov E., Rizvanov A., Khaiboullina S. (2017). Elevated Levels of Proinflammatory Cytokines in Cerebrospinal Fluid of Multiple Sclerosis Patients. Front. Immunol..

[59] Shi Z.R., Mabuchi T., Riutta S.J., Wu X., Peterson F.C., Volkman B.F., Hwang S.T. (2023). The Chemokine, CCL20, and Its Receptor, CCR6, in the Pathogenesis and Treatment of Psoriasis and Psoriatic Arthritis. J. Psoriasis Psoriatic Arthritis.

[60] Dieu-Nosjean M.C., Massacrier C., Vanbervliet B., Fridman W.H., Caux C. (2001). IL-10 induces CCR6 expression during Langerhans cell development while IL-4 and IFN-gamma suppress it. J. Immunol..

[61] Nichols M.R., St-Pierre M.K., Wendeln A.C., Makoni N.J., Gouwens L.K., Garrad E.C., Sohrabi M., Neher J.J., Tremblay M.E., Combs C.K. (2019). Inflammatory mechanisms in neurodegeneration. J. Neurochem..

[62] Hu W.T., Chen-Plotkin A., Arnold S.E., Grossman M., Clark C.M., Shaw L.M., Pickering E., Kuhn M., Chen Y., McCluskey L. (2010). Novel CSF biomarkers for Alzheimer’s disease and mild cognitive impairment. Acta Neuropathol..

[63] Bekker P., Ebsworth K., Walters M.J., Berahovich R.D., Ertl L.S., Charvat T.T., Punna S., Powers J.P., Campbell J.J., Sullivan T.J. (2015). CCR9 Antagonists in the Treatment of Ulcerative Colitis. Mediat. Inflamm..

[64] Wendt E., Keshav S. (2015). CCR9 antagonism: Potential in the treatment of Inflammatory Bowel Disease. Clin. Exp. Gastroenterol..

[65] Keshav S., Vaňásek T., Niv Y., Petryka R., Howaldt S., Bafutto M., Rácz I., Hetzel D., Nielsen O.H., Vermeire S. (2013). Prospective Randomized Oral-Therapy Evaluation in Crohn’s Disease Trial-1 PROTECT-1 Study Group. A randomized controlled trial of the efficacy and safety of CCX282-B, an orally-administered blocker of chemokine receptor CCR9, for patients with Crohn’s disease. PLoS ONE.

[66] Atanes P., Lee V., Huang G.C., Persaud S.J. (2020). The role of the CCL25-CCR9 axis in beta-cell function: Potential for therapeutic intervention in type 2 diabetes. Metabolism.

[67] Sędzikowska A., Szablewski L. (2021). Insulin and Insulin Resistance in Alzheimer’s Disease. Int. J. Mol. Sci..

[68] Da Mesquita S., Herz J., Wall M., Dykstra T., de Lima K.A., Norris G.T., Dabhi N., Kennedy T., Baker W., Kipnis J. (2021). Aging-associated deficit in CCR7 is linked to worsened glymphatic function, cognition, neuroinflammation, and β-amyloid pathology. Sci. Adventure.

[69] Jaehne E.J., Baune B.T. (2014). Effects of chemokine receptor signalling on cognition-like, emotion-like and sociability behaviours of CCR6 and CCR7 knockout mice. Behav. Brain Res..

[70] Gowhari Shabgah A., Al-Obaidi Z.M.J., Sulaiman Rahman H., Kamal Abdelbasset W., Suksatan W., Bokov D.O., Thangavelu L., Turki Jalil A., Jadidi-Niaragh F., Mohammadi H. (2022). Does CCL19 act as a double-edged sword in cancer development?. Clin. Exp. Immunol..

[71] Krumbholz M., Theil D., Steinmeyer F., Cepok S., Hemmer B., Hofbauer M., Farina C., Derfuss T., Junker A., Arzberger T. (2007). CCL19 is constitutively expressed in the CNS, up-regulated in neuroinflammation, active and also inactive multiple sclerosis lesions. J. Neuroimmunol..

[72] Simonetti O., Goteri G., Lucarini G., Filosa A., Pieramici T., Rubini C., Biagini G., Offidani A. (2006). Potential role of CCL27 and CCR10 expression in melanoma progression and immune escape. Eur. J. Cancer.

[73] Liu Y., Xiao A., Zhang B. (2021). CCR10/CCL27 crosstalk regulates cell metastasis via PI3K-Akt signaling axis in non-small-cell lung cancer. Am. J. Transl. Res..

