# Supplementary figures and images for: Inflammatory Mediators of Alzheimer’s Disease Characterized in a Mouse Model (APP/PS1)
Source: NeuroSci. 2026 Feb 6;7(1):23. doi: 10.3390/neurosci7010023 (PMC12921763; doi:10.3390/neurosci7010023)

## Slide 1
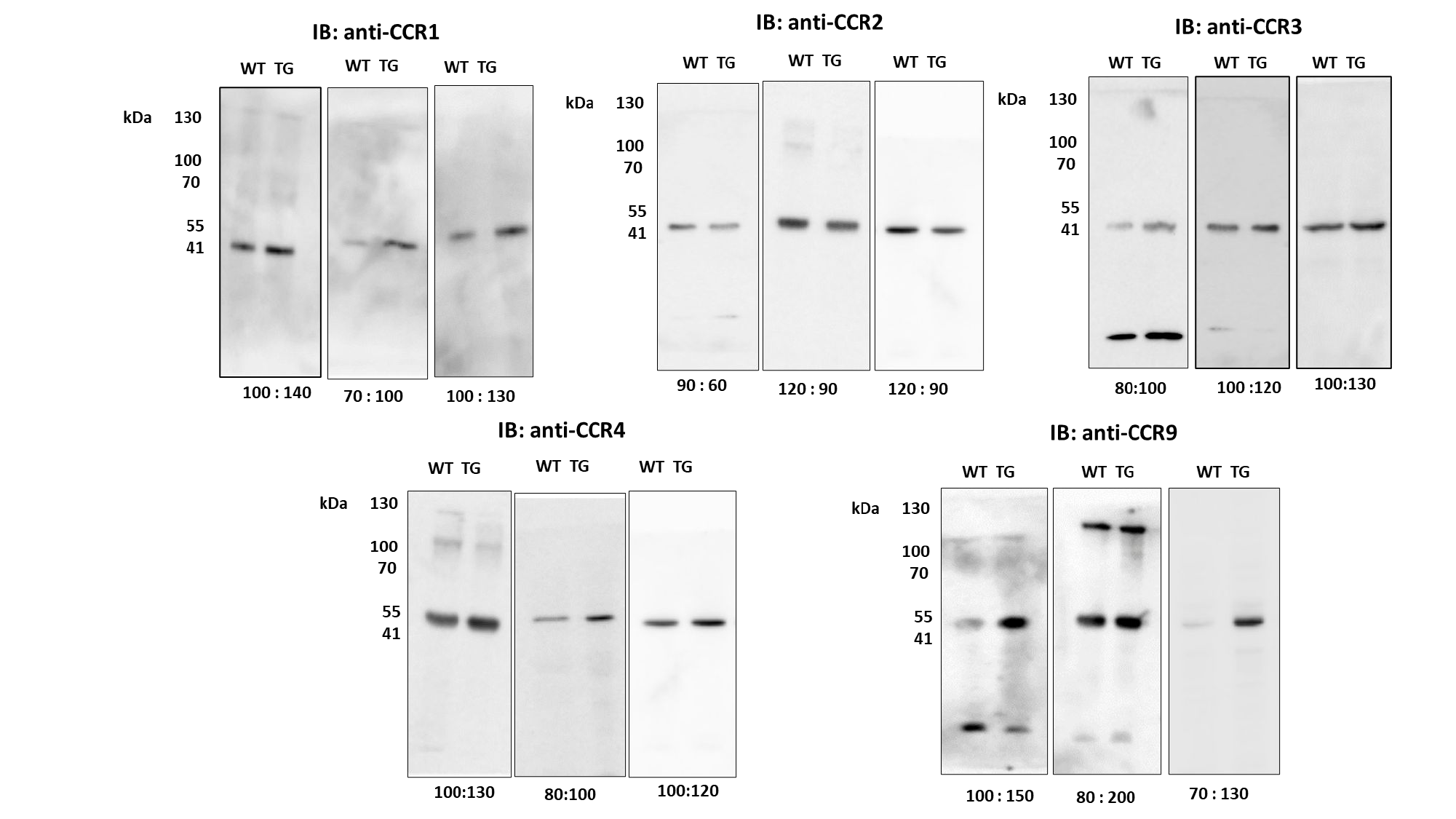

## Slide 2
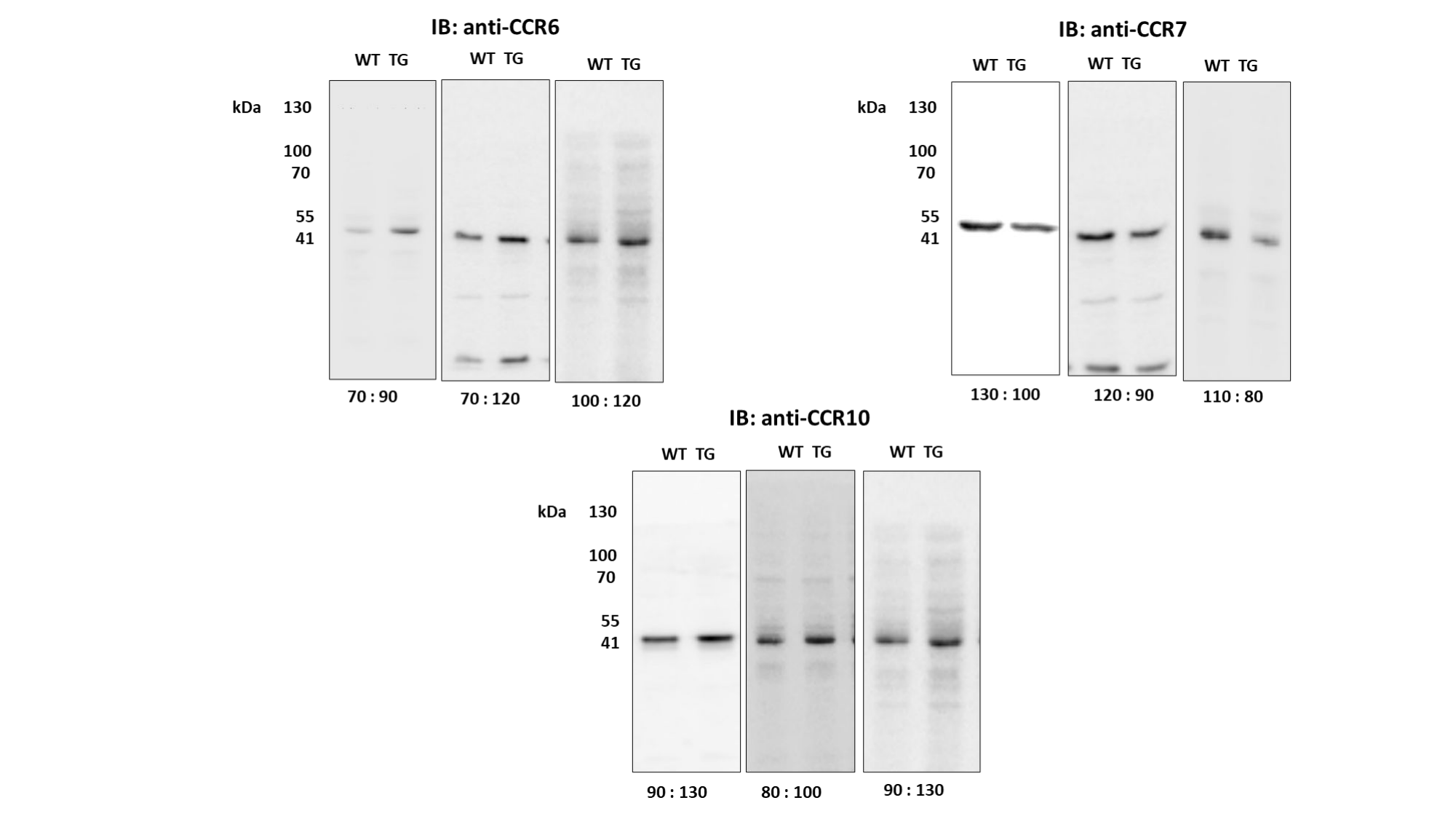

## Slide 3
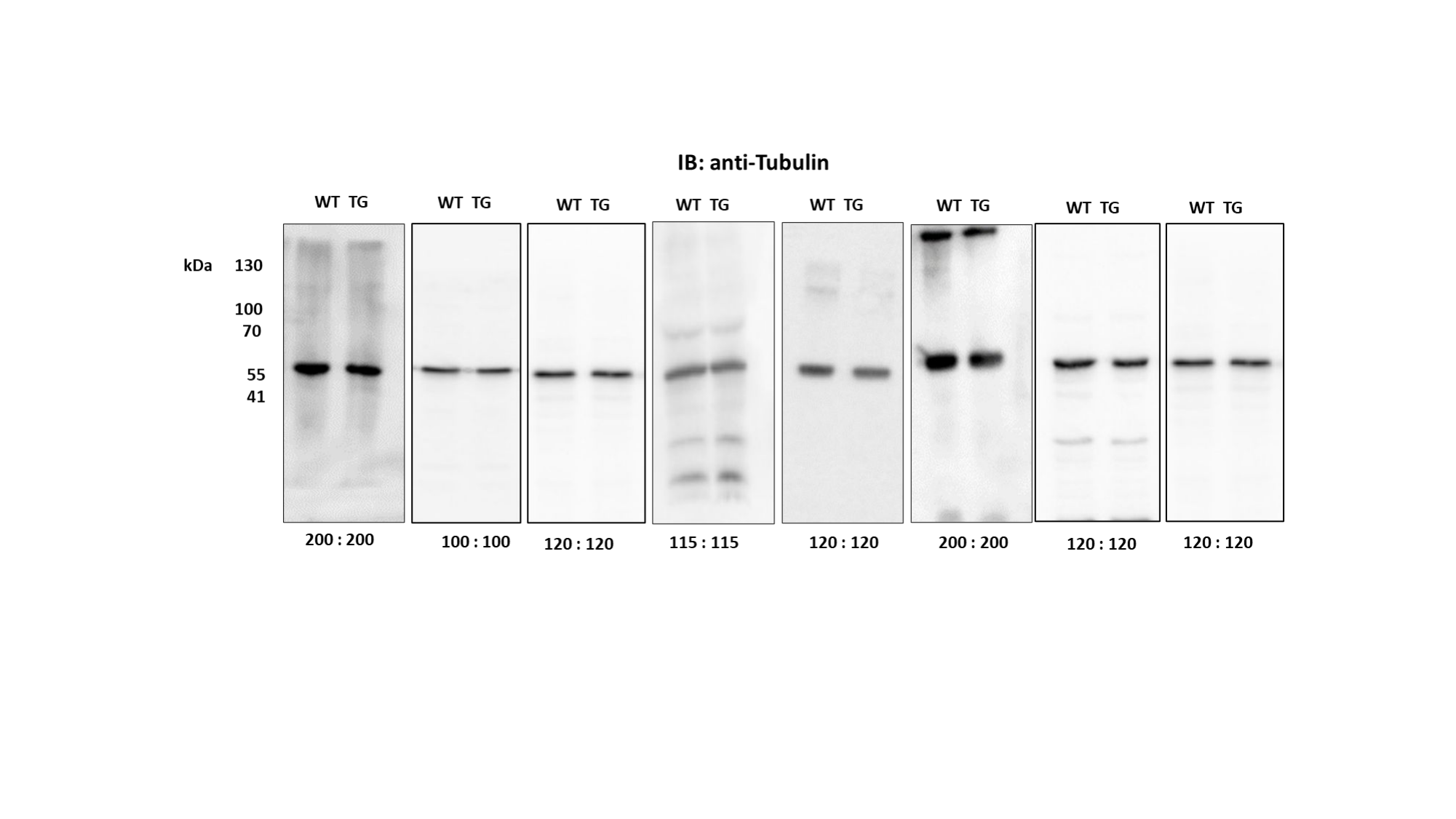

Supplement: Supplementary file 1 [file neurosci-07-00023-s001.zip › neurosci-4122712-supplementary.pptx]
